# All-optical design for inherently energy-conserving reversible gates and circuits

**DOI:** 10.1038/ncomms11424

**Published:** 2016-04-26

**Authors:** Eyal Cohen, Shlomi Dolev, Michael Rosenblit

**Affiliations:** 1 Department of Computer Science, Ben Gurion University of the Negev, 653 Be'er, Sheva 84105, Israel; 2 Ilze Katz Institute for Nanoscale Science and Technology, Ben Gurion University of the Negev, 653 Be'er, Sheva 84105, Israel

## Abstract

As energy efficiency becomes a paramount issue in this day and age, reversible computing may serve as a critical step towards energy conservation in information technology. The inputs of reversible computing elements define the outputs and vice versa. Some reversible gates such as the Fredkin gate are also universal; that is, they may be used to produce any logic operation. It is possible to find physical representations for the information, so that when processed with reversible logic, the energy of the output is equal to the energy of the input. It is suggested that there may be devices that will do that without applying any additional power. Here, we present a formalism that may be used to produce any reversible logic gate. We implement this method over an optical design of the Fredkin gate, which utilizes only optical elements that inherently conserve energy.

## Abstract (web summary)

Reversible gates, like Fredkin gates, may be useful for energy conservation efforts. Cohen *et al*. present a formalism that may be used to produce any reversible logic. This method is implemented over an optical design of the Fredkin gate which utilizes only optical elements that inherently conserve energy.

## Graphical abstract

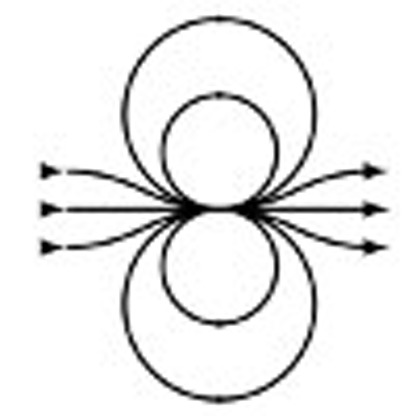

One of the greatest challenges in computing is to reduce the energy consumption, not only for the sake of reducing the tremendous amount of electric power used by the computer-based industry (for example, Google, Amazon or Facebook) but also to enable computing devices to operate at a higher frequency without melting as a result of the extra heat. A radical change in computer design may possibly lead to the crucial breakthrough needed for achieving more energy-efficient information processing. One promising direction for a breakthrough is to implement an all-optical^1^ universal reversible gate, such as the Fredkin gate^2^, and then use the Fredkin gate implementation as a building block in reversible circuits, thus overcoming this significant challenge.

The Fredkin gate is a universal reversible logic gate. Its universality means that any logic operation can be produced using only Fredkin gates. It has been suggested that reversible gates may preserve energy together with the data and reversible devices may be built entirely with energy-conserving elements^3^,^4^. These elements include a network of directional couplers and controlled nonlinear phased modulators. While the ability to build energy-conserving reversible gates has been discussed, no proof was given for the feasibility of these devices. In addition, no mathematical model has been presented which describes how to design such devices. Therefore, the search for a universal, energy-conserving, reversible logic device is still crucial for reversible computing and its beneficial prospects.

Research in the field of reversible computing is focused on two major aspects which include the implementation of reversible gates on one hand, and their usage for large-scale circuits on the other.

Extensive work has been done trying to utilize the Fredkin gate to perform simple or complex computing primitives. The assumption is that an ideal Fredkin gate is available and used to build efficient circuits. For example, a paradigm named ‘Directed Logic'^5^ is presented as an energy-efficient computation model used for Boolean gates. Later on, directed logic was used to build automata and circuits^6^.

The other major aspect in the research of reversible computing suggests implementations for the Fredkin gate. Publications differ in their technological paradigms. Therefore, we will refer to them as the ‘electronic approach', the ‘all-optical approach' and the ‘hybrid electro-optic approach'. Since electronic and hybrid devices rely on transistors and similar semiconductors paradigms they suffer from inherent energy loss.

Previous publications in the scope of the electronic approach suggested complementary metal-oxide semiconductor (CMOS) implementations^7^. The designs focus on an effort to minimize the energy loss and measure it on every logical state of the gate.

The hybrid electro-optic approach suggests that data and control signals may be partly optical and partly electronic. For example, implementations for ‘directed logic' are suggested^8^,^9^,^10^, where an electric control may be used for switching the two inputs to the appropriate two outputs. The control is based on manipulation of the resonance of a ring resonator by a silicon p-i-n junction built over the cross-section of the ring^11^,^12^. This approach requires the conversion of optical data to electronic controls, or electronic signals to optical ones.

The last approach suggests an all-optical approach, where all inputs and controls are optical. For example, it is suggested that light projection may be used to mechanically manipulate the positioning of molecules^13^. This minute manipulation may be very significant if it is done over a ring or a disc resonator. The movement may tune or detune the resonator and, in essence, implement a switch. This design is still not energetically efficient, since the optical energy is transformed into mechanical energy.

Other examples involve utilizing an effect called four-wave mixing (FWM). Under certain conditions in a nonlinear medium, a strong continuous wave signal on one wavelength may switch signals between two other wavelengths. When the continuous wave is absent no manipulation is done, and the signals continue to propagate with negligible modification. This technique is used to implement a Fredkin gate over the wavelength space^14^. A very high intensity is needed for the continuous wave, in order to exhibit the expected wavelength conversion, which also suffers from losses such as harmonics generation. However, it is possible to lower these losses^15^ by utilizing coupled-resonator optical waveguides in order to enhance the FWM interaction. The main issue with FWM implementations is that all signals propagate in the same waveguide which requires additional separation, amplification or wavelength conversion in order to cascade the gates.

A different approach is to attempt to utilize quantum optics in order to produce quantum optical gates^16^,^17^. Although these devices are relatively simple in design, they require single-photon sources. While single-photon sources are becoming available^18^, these devices are limited to work only with single-photon sources, making them difficult to integrate with non-quantum optical elements.

It should be noted that from a logic point of view, reversible gates are quantum gates and quantum gates are reversible gates. The two fields share similar concepts and mathematical approaches. However, while quantum gates require quantum implementations, reversible gates are not limited by that requirement, and our formalism and implementation do not refer necessarily to quantum mechanics or quantum implementations.

Here, we present a design for reversible gates built entirely from energy-conserving elements. First, we present our formalism, that utilizes linear and nonlinear unitary transformations in relation to conserving optical elements such as directional couplers. This formalism may be used by designers to design any reversible logic. Next, we use this formalism in order to design a Fredkin gate. We also simulate the different optical elements used such as directional couplers and graphene-induced silicon waveguide showing negligible energy loss. These results are later used to simulate our design of the Fredkin gate. All this effort was made while taking into account further circuit design. For example, making sure that all inputs and outputs are interchangeable without power or wavelength modification. Also, it will permit any cascading design of the circuits.

## Results

### Optical elements

When considering a computational machine, theoretical values need to be associated with a physical value. In an optical implementation for example, values used will refer to an amplitude of an electric field. The energy hiding in this field is proportional to the square of the magnitude of the electric field. To preserve the overall energy, all values may interact using unitary transformations which preserve the sum over the squares of the magnitudes.

Supplementary Notes 1 and 2 present a few basic available building blocks that manipulate the optical signals in a unitary fashion while Supplementary Fig. 1 demonstrates a representation of the relevant parameters of a general directional coupler. We are going to use these building blocks in order to design our devices. These elements include a linear waveguide which is used to introduce a phase to a signal, and a nonlinear Kerr-induced waveguide, which is used to introduce a nonlinear phase to a signal. Also, we will explain how a directional coupler or a Mach–Zehnder coupler performs a linear unitary transformation on two signals, while an array of such couplers may be used to perform a linear unitary transformation over a set of signals.

### Unitary transform formalism

We would like to propose a general theory that allows the design and building of devices that solve a specific reversible logic gate. For example, we will explain the steps needed to design the reversible Fredkin gate. A truth table of the Fredkin gate is given in Table 1. The inputs of the device are considered in a straightforward manner, where each input channel represents a logical input value of the gate. In binary gates, each of these inputs may hold two complex values *E*_0_ and *E*_1_, which are amplitudes that would represent the states 0 and 1, respectively. Similarly, the outputs of the device may also hold the same values as those determined by the gate logic.

Given a state of the gate, we can arrange the inputs for each input channel in a column vector. The different vectors may be arranged side-by-side to build a matrix. This will be the input matrix, which we will denote with *A*. Similarly, we can arrange the outputs for each output channel in a column vector and arrange these vectors side-by-side to build an output matrix, which is denoted by *B*.

Our goal is that when the device receives an input that is a column of *A*, it will produce an output corresponding to an appropriate column of *B*. The number of columns on both matrices is the number of states the gate supports. The number of rows in *A* is the number of input channels and the number of rows in *B* is the number of output channels. Notice that if the gate is reversible, the number of input and output channels is the same, and so are the dimensions of *A* and *B*.

If we can find a unitary matrix *M* that satisfies *B*=*MA*, we can stop here and build the device using a coupling array that decomposes the matrix. Details on unitary matrix decomposition is given in Supplementary Notes 3 and 5, while Supplementary Fig. 2 demonstrates a coupling array. However, generally, such unitary matrices may not be found, particularly in the case of Fredkin gate, merely because of the fact that the number of columns is greater than the number of rows of *A* and *B*. That would mean that the dimensions of *M* are too small and the degrees of freedom are not enough to solve the equation.

To overcome this problem, we may add rows to both *A* and *B*. We denote the matrix added to *A* with *F*′ and the matrix added to *B* with *F*. *F* is part of the product of the multiplication, while after a small modification it will turn into *F*′, which is then used as part of the multiplier. The transformation from *F* to *F*′ is done with no loss. Hence, the prime represents a little modification to *F*.

In other words, we are looking for a unitary matrix *M* that solves:


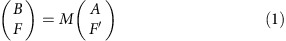


**Figure 1:**
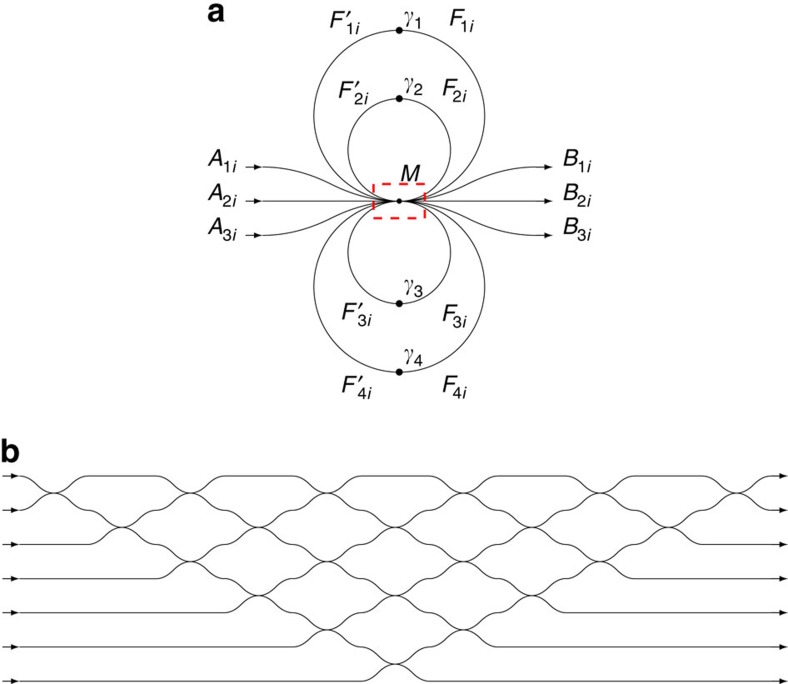
A representation of a three channel gate. (**a**) This example shows three inputs denoted by *A*, and three outputs denoted by *B*. Four more channels are used for feedback, and denoted by *F* and *F*′. The coupling is between seven channels and denoted by *M* and a red broken box. (**b**) A zoom on the coupling *M*. This coupling array couples seven channels: the inputs are three *A* channels and four *F*′ channels. Similarly, the outputs are three *B* channels and four *F* channels. This coupling is decomposed to 21 couplings between adjacent channels. When *F*′ is added as an input for the coupling, *F* is added to the output of the coupling. Each *F* is then manipulated with an appropriate nonlinear element denoted by γ, which transforms it to *F*′. Then, *F*′ is fed back to the system. The column index *i* represents different states.

A schematic design of the presented setup is given in Fig. 1. The design illustrated presents three inputs, three outputs and four feedback loops with a nonlinear element. Note that all presented elements relate to the matrices *A*, *B*, *F* and *F*′, which hold the electric field over each channel at different states. The row index of the matrices defines the channel and the column index represents the state. The gate illustrated in Fig. 1a exhibits coupling between seven channels denoted by *M*. This seven-channel coupling is designed with 21 adjacent couplings illustrated in Fig. 1b. Also, each *F* channel is manipulated through a nonlinear effect denoted by γ to become *F*′.

Note that in Fig. 1b, there is no notation for the different channels, it is not described which inputs hold the *A* channels or the *F*′ channels or which outputs hold the *B* channels or the *F* channels. We are free to choose any permutation of these channels as this permutation can be described by a unitary matrix that may be integrated as part of *M*. A permutation should be chosen such that the decomposition of *M* will be the easiest to manufacture, taking into account manufacturing properties and limitations.

Two questions are raised by adding the rows of *F*′ and *F* to *A* and *B*. The first regards how many rows need to be added to *A* or *B* in the form of *F*′ and *F* respectively. The answer is that we cannot predict the dimensions of *F*, and each case should be investigated for its properties. The second regards whether it is possible to have feedback with no nonlinear elements, or is the nonlinearity obligatory.

We can try to use the relation between *A*, *B*, *F* and *F*′ through the unitary matrix *M* and get:


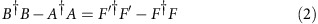


Note that in this expression *M* is eliminated. The left-hand side of the equation is determined only by the state representation of an examined gate. Also, note that if the gate is conserving energy for every state, the diagonal of the left-hand side is filled with zeros. Moreover, if some elements of the left-hand side are not zero, this means that a nonlinear interaction must come into play in order to manipulate *F* to be *F*′, such that this condition is satisfied.

Note, however, that this equation was derived from equation (1), but it holds less information. A solution for equation (1) is therefore more inclusive and we are going to focus on it.

While different solutions for equation (1) may be found, it does not mean that all these solutions may converge to a desired output given an input. In fact, it only means that the desired states are steady states. These states may also be weak steady states, that might diverge after a short time period. Supplementary Note 4 discusses the issue of convergence and its time period. Supplementary Notes 6 and 7 also discusses different representations for logical data using optics, giving examples of the XOR and Fredkin gates.

### Numerical solution for Fredkin gate

**Figure 2:**
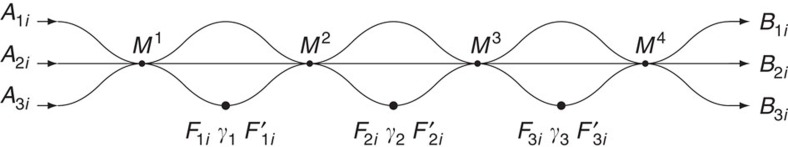
An implementation of the Fredkin gate where feedbacks are eliminated. This design uses four coupling nodes. All nodes couple three channels. Three channels are used for nonlinear manipulation and denoted by *F* and *F*′. Each *F* is then manipulated with an appropriate nonlinear element denoted by γ, which transforms it to *F*′. The column index *i* represents different states.

The terms and conditions formulated were implemented in Matlab, where numerical solutions were found for the different elements of the Fredkin gate. A solution for equation (1) was found, where *M* is later decomposed to an array of four 3 × 3 matrices, while the design utilizes three nonlinear elements. This design is given in Fig. 2. Note that feedback was eliminated with this design, which allows the interaction of pulsed light and not necessarily based on continuous wave. The derivation of this design is further discussed in the methods section. The minimal propagation time through this element is proportional to the sum of the gamma values which was minimized to *S*_*γ*_=12.5. Additional information about the derivation of this solution is given in Supplementary Note 8, while Supplementary Fig. 3 shows an intermediate stage in the decomposition of the design.

### Optical elements

Finite-difference-time-domain (FDTD) simulations are done over simple elements that include a symmetric waveguide coupler and a waveguide infused with a Kerr nonlinear element. Subsequently, these elements are used as building blocks for photonic integrated circuits.

**Figure 3:**
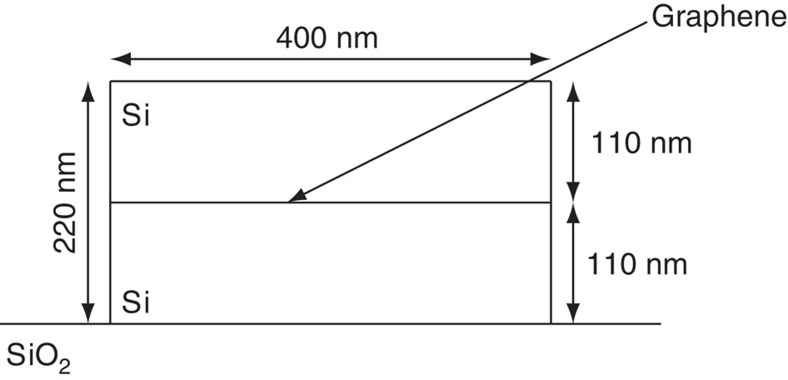
An illustration of a waveguide cross-section. The waveguide is rectangular silicon over a glass substrate. The width of the waveguide is 400 nm and its height is 220 nm. When needed, a graphene sheet is included into the waveguide centred at the middle with a height (or thickness) of 1 nm. The graphene is included only when the Kerr effect is needed, while it is not included otherwise.

All the simulations assumed rectangular waveguides of silicon (Si) layered over glass substrate. The waveguide had a width of 400 nm and height of 220 nm. The simulations were done with a wavelength of *λ*=1.5 μm, since it exhibits negligible absorption in Si and low bend loss in single-mode waveguides when utilizing the recent advances in CMOS production^19^,^20^. Note, however, that Si single-mode waveguides produced utilizing these methods still exhibit a relatively high-propagation loss of 0.3*db* cm^−1^. In our discussion, we will remain with typical lengths that are overall smaller than 1 cm. Also, we may consider using different materials, resulting in a lower loss. All simulations refer to the fundamental propagating mode that this waveguide can support. An illustration of the cross-section is given in Fig. 3.

Note that this waveguide profile supports only single-mode propagation. The sources used in the simulation stimulate only this mode, while the monitors that read the corresponding outputs compare their results to this mode. It is assumed that only this mode may propagate and all discussions refer to this mode.

While many simulations, fabrications and tests on directional couplers and Mach–Zehnder interferometers were done and produced significant results, we are still required to produce our own design and results. First, we intend to investigate the losses presented by these elements. Moreover, we choose a specific wavelength and a specific cross-section throughout the design for all waveguides. These waveguides should match the simulations done for the Kerr-induced waveguide. The desired data and parameters may be problematic to obtain based solely on past literature; hence, these elements must be simulated again. Details of these simulations are given in the methods section and Supplementary Note 9. In addition Supplementary Fig. 4 shows the schematic design of the coupler used in our simulations.

A set of directional couplers were simulated to characterize their properties. An example of a propagation process in our design of the directional coupler is given on Supplementary Fig. 5. The results show a loss lower than 0.3% in the energy, or −0.013*db* for all the range of elements simulated. Supplementary Fig. 6 illustrates the derived coupling values as a function of the bend radius.

This element is later used to build a Mach–Zehnder interferometer by integrating two 50% directional couplers. The results for the Mach–Zehnder interferometer show a loss lower than 0.3% in the energy. An example of a propagation process in our design of the Mach–Zhender coupler is given on Supplementary Fig. 7.

**Figure 4:**
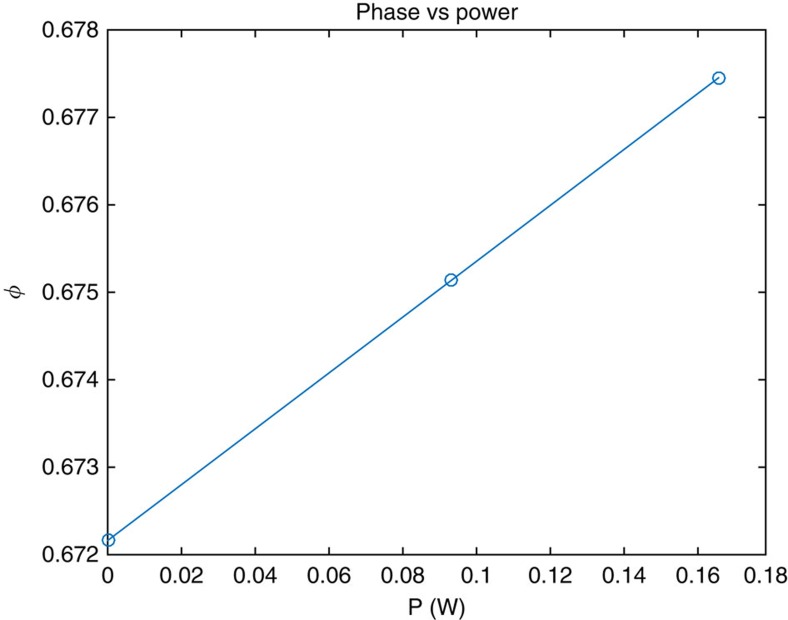
Phase as a function of the power. A minor phase response is observed when the power propagating through the waveguide is increased. The waveguide includes graphene with a thickness of 1 nm. The length of the Kerr element is 5 μm.

A graphene infused waveguide was also simulated. The length chosen for the Kerr element simulation was *L*=5 μm. The results show a negligible loss, lower than 0.07% in the energy, or −0.003*db*. The phase response is given in Fig. 4. It shows a relatively high response giving:


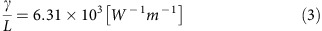


As explained earlier, a sum of the nonlinear values was calculated as *S*_*γ*_=12.5. This result corresponds to a length of *L*=2 *mm* and a converging time of *T*=2 × 10^−11^ s when using 1 W sources. A convergence energy *E*_*c*_=2 × 10^−11^ J. Note of course that a faster system convergence may be achieved with higher power and designing shorter elements. Also, low-power devices may be produced with longer elements at the expense of the system convergence time.

### Integrated circuit

The elements investigated in the FDTD simulation were now used for an integrated circuit simulation. The allows incorporating results acquired with ‘Lumerical FDTD Solutions' for a specific basic optical element. These elements may be inter-connected into a simulation of one integrated device. It also provides many built in optical elements such as sources, detectors, Mach–Zehnder coupler, phase and delay lines which may be used together with customized scripted elements, and other integrated compiled elements.

**Figure 5:**
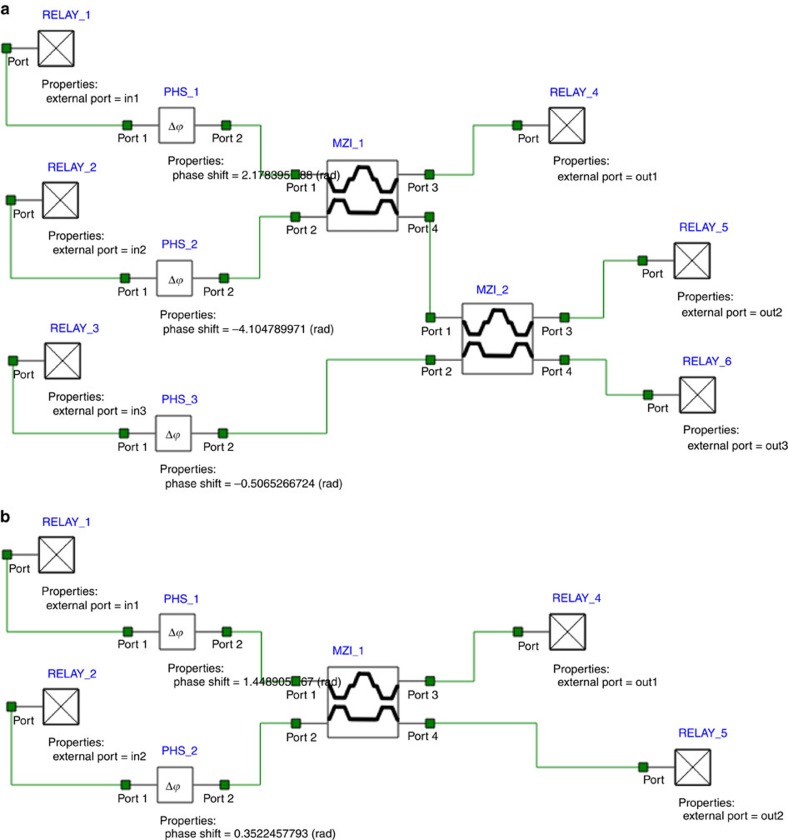
Compiled elements that perform a unitary transformation. (**a**) An expansion of the compiled element ‘mzi-comp' that includes two MZI couplers and three phase elements. (**b**) An expansion of the compiled element ‘mzi-ex' that includes one MZI coupler and two phase elements.

First, in order to simplify the scheme of the design we compile and script a 2 × 2 unitary transformation by integrating a Mach–Zehnder coupler with phase elements into one compiled element titled ‘mzi-ex'. The script selects the proper values for the coupler and phase elements such that the compiled element performs a user defined unitary transformation. Similarly, we compile and script a 3 × 3 unitary transformation by integrating two Mach–Zehnder couplers with phase elements into one compiled element titled ‘mzi-comp'. These elements are displayed in Fig. 5. All couplings were achieved with Mach–Zehnder coupler elements with two 50% couplers and typical phase lengths of 15 μm.

**Figure 6:**
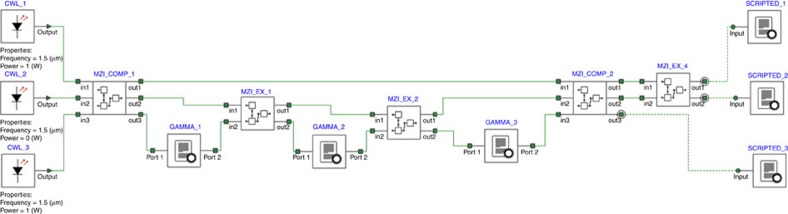
Schematics of the device suggested. This scheme uses seven Mach–Zehnder couplers, and phase shift elements. This scheme uses compiled elements ‘mzi-comp' and ‘mzi-ex' in order to simplify the display of the design. Also, the nonlinear Kerr elements were created and are labelled ‘gamma'.

The schematics of the Fredkin gate device are given in Fig. 6. These schematics include the compiled elements from Fig. 5. Three sources were used as input with the power values of zero or 1 W representing the logical 0 and 1. The nonlinear Kerr elements were scripted to produce a phase shift proportional to the power. Output values were collected with scripted elements (titled ‘scripted') allowing us to collect phase information together with the intensity.

The integrated circuit was tested with all possible inputs of the Fredkin gate. First, a simulation was done using ideal elements with no loss. The results show almost no deviation from the desired output.

**Figure 7:**
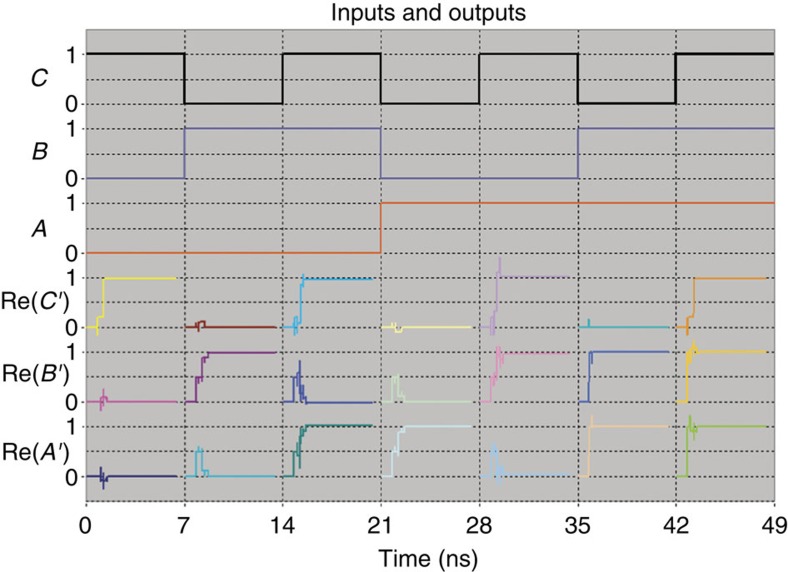
Timing sequences of the Fredkin gate. Input values and output results are presented across time. The three input values *A*, *B* and *C* are shown together with the real values of the outputs *A*′, *B*′ and *C*′. As expected from a Fredkin gate, the value of the output *C*′ is very similar to the value of the input *C*. When *C*=0, the values of *A*′ and *B*′ are similar to the values of *A* and *B* respectively. When *C*=1, the values of *A*′ and *B*′ are similar to the values of *B* and *A*, respectively. Note the time delay of the outputs as a result of the propagation delay over the optical elements.

Next, losses were introduced, according to the results of the FDTD simulations, where 0.3% of energy loss was applied over all Mach–Zehnder interferometers. The result of the integrated circuit simulation are displayed on Fig. 7 which displays the timing sequence for the different possible signals of the Fredkin gate where *A*, *B* and *C* are three input signals and *A*′, *B*′ and *C*′ are the three output signals. These results exhibit a small deviation from the desired values. The highest error introduced to the results occurred on the *ABC*=101 state, where the normalized result of the *B*′ channel was *E*_*B*′_=0.961–0.044*i* instead of the value 1, which is a 6% deviation over the complex plane.

## Discussion

A formalism for the design of reversible circuits was presented. This formalism utilizes only unitary transformations. Linear unitary transformations were represented by a unitary matrix. The definition of unitary transformations was extended to nonlinear transformations by maintaining that the sum over all the squares of the magnitudes is preserved.

The formalism suggested provides a theoretical condition over the reversible gate truth table, to determine if the specific logic may be implemented only by linear elements, or whether it is required to use nonlinear elements.

Optical elements that maintain these unitary properties were presented and tested. A directional coupler or a Mach–Zehnder coupler are used to represent a 2 × 2 unitary matrix transformation. An array of these couplers may be used for a general unitary matrix transformation.

Also, a Kerr-induced waveguide was simulated. By using graphene as a Kerr material, the effect becomes very strong and shorter elements may be used. This element provides the nonlinear unitary transformation by inducing self phase modulation. Still, since Kerr interactions are usually weak the length needed for these elements is relatively high compared with other elements in the system. This length will determine the propagation time needed in order to achieve the required result.

Simulation over all optical elements show a very low loss, which is almost negligible. This demonstrates the superiority of optical elements in energy efficiency.

We used the formalism presented in order to determine the needed elements for a Fredkin gate. We tried to minimize the length of the nonlinear elements by minimizing the sum over the nonlinear values *S*_*γ*_. We also managed to eliminate feedback loops in the design, allowing the usage of pulsed light and not only continuous waves.

The integrated circuit simulation provided a promising result. The 6% deviation will still allow a cascading usage of the device in larger scale logic devices. Assuming the worst-case scenario where a 6% deviation is accumulated in a geometric progression, it is still possible to cascade the device five times before corrections with thresholds are needed.

This 6% deviation in the amplitude translates to a 7.5% energy loss. If we modify the device to support short pulses, a 1 W source with a pulse time of 50 fs is 10 wavelengths long, and it holds an energy of 50 fJ. The loss in that case is lower than 4 fJ, where we may assume that most of it probably propagates in the wrong channel rather than heating the system. A lower power source may be used to lower the loss even further; however, this will demand a longer propagation in the nonlinear element. Hence a lower power and lower loss may be achieved at the expense of a longer delay.

Also, this deviation may be lowered by modifying the numerical solution. This simulation was intended to assess the deviation of the output from an ideal device given losses. The results showed quasi-random deviation from the desired output. However, the numerical solution considered only ideal elements, and it may be modified to consider losses, such that the outputs will exhibit the same, yet minimal, power loss. These in turn may be used for other Fredkin gates that consider a lower input power as the normalized logical values.

We presented an optical architecture for reversible logic that does not dissipate heat and, while converged to a state, does not waste energy. The new mathematical framework provided here can be implemented for any reversible logic gate. The design was based on three basic energy-conserving elements: linear phase; coupling; and nonlinear manipulation.

The design is not limited to conventional optics, as different physical phenomena may be used. For example, electron optics may be considered. Another physical approach may suggest that the different inputs may be held on the same channel but on different wavelengths. Coupling between different wavelengths is possible through FWM, which was demonstrated efficiently over graphene^21^.

The device only utilizes elements that conserve energy, namely waveguides and conserving couplers as linear elements and Kerr effect materials for nonlinear manipulations. The linear coupling may be achieved with different technologies such as a mode-waveguide coupler, or Mach–Zehnder coupling. The Kerr effect was chosen since it is energy-conserving, is widely available with different materials, and is easily formulated. However, the Kerr effect may be replaced by a different nonlinear, energy-conserving effect. Different effects and materials may exhibit significantly stronger interactions, which would allow lower energy loss and higher working frequency.

While converging, the wasted energy is not transformed to heat but rather is removed from the system through the output ports. This energy may be reused. For example, it may be redirected back to induce population inversion which may be used at a laser source.

For ease of manufacturing, a small number of couplings should be used. In our case, the proposed Fredkin gate utilized only seven couplings. A relatively long time is needed to allow propagation through the nonlinear element. This is a result of the extremely weak interaction of the Kerr effect. To achieve better performance, a shorter wavelength should be used. Better performance may be achieved by the discovery and usage of materials with stronger Kerr interactions. Stronger Kerr interactions may be available with future investigation of graphene. It may even be possible to use a totally different effect for the nonlinear interaction.

The example given for the Fredkin gate used a binary representation for each input or output channel. It may be possible to design gates where each channel may hold more than two values. These values may also be complex.

Note that states of the device were defined by a complex value for the electric field and the Kerr effect was dependent on a continuous wave. Future research may try to redesign the device such that it may support short pulses of light. This may be done by carefully choosing the lengths of the elements, or the optical lengths, and allowing signals that interact with each other to arrive and interact with each other at the appropriate point in time and space. By using pulses, however, special attention should be given to the shape of the pulse when dispersion and the Kerr effect may destroy the shape and introduce undesired noise. It may be possible that a soliton solution may be found while considering the presence of these effects.

Although our device introduces long time delay, a device that supports pulses may not necessarily suffer from bandwidth loss. This is because the device may be used asynchronously, while interaction with the pulses does not modify the shape of the pulse.

This device may serve as an intermediate step towards reversible optical quantum computing. The next step may be to design the devices that incorporate energy-efficient elements together with elements that are governed by quantum effects, while supporting one-photon interaction together with two-photon, three-photon or four-photon interactions.

Another approach may use the formalism and design described and integrate it with a semiconductor optical amplifier only to compensate for the minor optical losses. This may lead to a simpler design while maintaining a very low-power consumption.

## Methods

### Fabrication feasibility

When we deal with the available building blocks, we have to remember that we want to build devices with negligible loss. In other words, the sum of the intensity over all inputs should be nearly the same as the sum of the intensity over all outputs.

Note that negligible loss is possible to maintain providing the advances in fabrication. An ‘ultra-high-Q' optical resonator has been presented^22^, featuring a design and fabrication process that managed to produce a toroid microcavity resonator on a chip with a Q factor in excess of 100 million. This suggests that loss is negligible and was eliminated by the advanced fabrication methods described.

The formalism presented deals with an ideal theory behind the device. Several parameters and assumptions were chosen and are given in Supplementary Note 10. In the results section, we tested different optical elements, to challenge these assumptions. We tested the feasibility of physically implementing this theory and ultimately demonstrated that these assumptions are almost entirely correct.

### Numerical solution

Matlab was used to solve equation 1. A solver was used to find the parameters of the matrix *M*, under the conditions that it is a unitary matrix. The matrix *M* was also confined to the convergence conditions given in Supplementary Note 4. The values of *F* are dependent on the values of *M*. The values of *F*′ were chosen according the relation 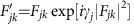, where *γ*_*j*_ is the scaled Kerr coefficient. This solver also provided the values of *γ*_*j*_ while optimizing the solutions such that the sum ∑_*j*_*γ*_*j*_ will be minimized.

Another Matlab script was used to decompose the matrix *M* into arrays of 2 × 2 unitary transformations. These values were later used in the simulation of the integrated device.

### Optical simulations

Simulations were done in two stages. First, the different optical elements were tested using ‘Lumerical FDTD solutions'. The simulations included directional couplers, Mach–Zehnder couplers, and Kerr elements. Details about the FDTD simulations of the different optical elements is given in Supplementary Note 9.

Later, a simulation of the integrated circuit using ‘Lumerical Interconnect'. ‘Lumerical Interconnect' allows incorporating results acquired with ‘Lumerical FDTD Solutions' for a specific basic optical element. ‘Lumerical Interconnect' also allows the scripting of its elements which was necessary for elements such as general unitary couplers, Kerr elements and the different detectors.

## Additional information

**How to cite this article:** Cohen, E. *et al*. All-optical design for inherently energy-conserving reversible gates and circuits. *Nat. Commun.* 7:11424 doi: 10.1038/ncomms11424 (2016).

## Supplementary Material

Supplementary Information Supplementary Figures 1-7, Supplementary Notes 1-10 and Supplementary References.

## Figures and Tables

**Table 1:** A truth table of the reversible Fredkin gate.

***C***	***X***_**1**_	***X***_**2**_	***C*****′**	***Y***_**1**_	***Y***_**2**_
0	0	0	0	0	0
0	0	1	0	0	1
0	1	0	0	1	0
0	1	1	0	1	1
1	0	0	1	0	0
1	0	1	1	1	0
1	1	0	1	0	1
1	1	1	1	1	1

*X*_1_, *X*_2_ and *C* represent three input bits, while *Y*_1_, *Y*_2_ and *C*′ represent three output bits.
