# Supplementary material for: All-optical design for inherently energy-conserving reversible gates and circuits
Source: Nat Commun. 2016 Apr 26;7:11424. doi: 10.1038/ncomms11424 (PMC4853429; doi:10.1038/ncomms11424)
Supplement: Supplementary Information — Supplementary Figures 1-7, Supplementary Notes 1-10 and Supplementary References. [file ncomms11424-s1.pdf]

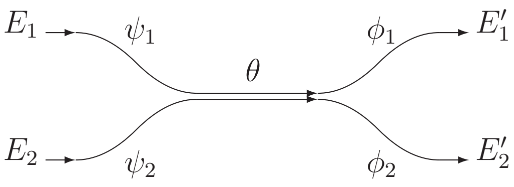

Supplementary Figure 1: A representation of a directional coupler. For the simplicity of the illustration, the width of the waveguides is reduced to lines where each line represent a dielectric waveguide. ψ_1 is a phase added to the first incoming channel E_1. ψ_2 is a phase added to the second incoming channel E_2. ϕ_1 is a phase added to the first outgoing channel E'_1. ϕ_2 is a phase added to the second outgoing channel E'_2. θ is the coupling angle that determines the coupling coefficient.

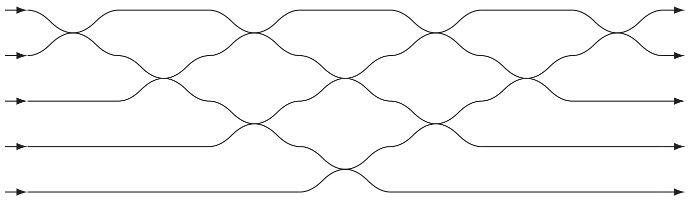

Supplementary Figure 2: A representation of five channels coupling. The coupling is done using 10 couplings between adjacent channels.

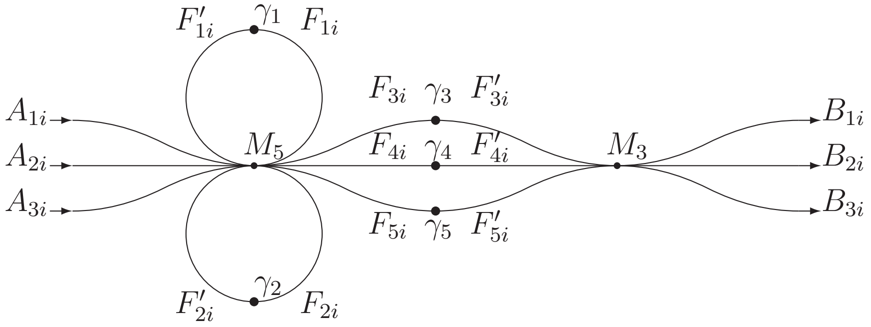

Supplementary Figure 3: A proposal for an implementation of the Fredkin gate. This design uses two coupling nodes. The first node couples 5 channels and is denoted by M_5. The second node couples 3 channels and is denoted by M_3. This example shows three inputs denoted by A, and three outputs denoted by B. Two channels are used for feedback and three channels are used for additional non-linear manipulation and denoted by F and F'. Each F is then manipulated with an appropriate non-linear element denoted by γ, which transforms it to F' and feeds it back to the system. The column index i represents different states.

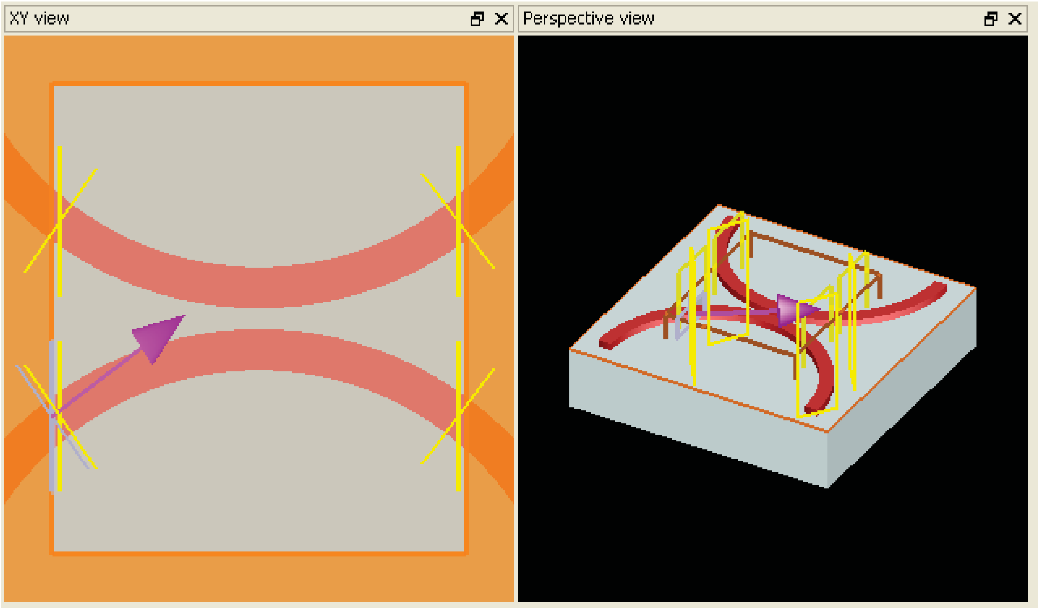

Supplementary Figure 4: Schematic design of a directional coupler. Two circular silicon waveguides are layered over glass. The two waveguides get very close to each other and a small gap is between them such that coupling is enabled. A source is placed over the bottom left port while four monitors are placed over all the output ports.

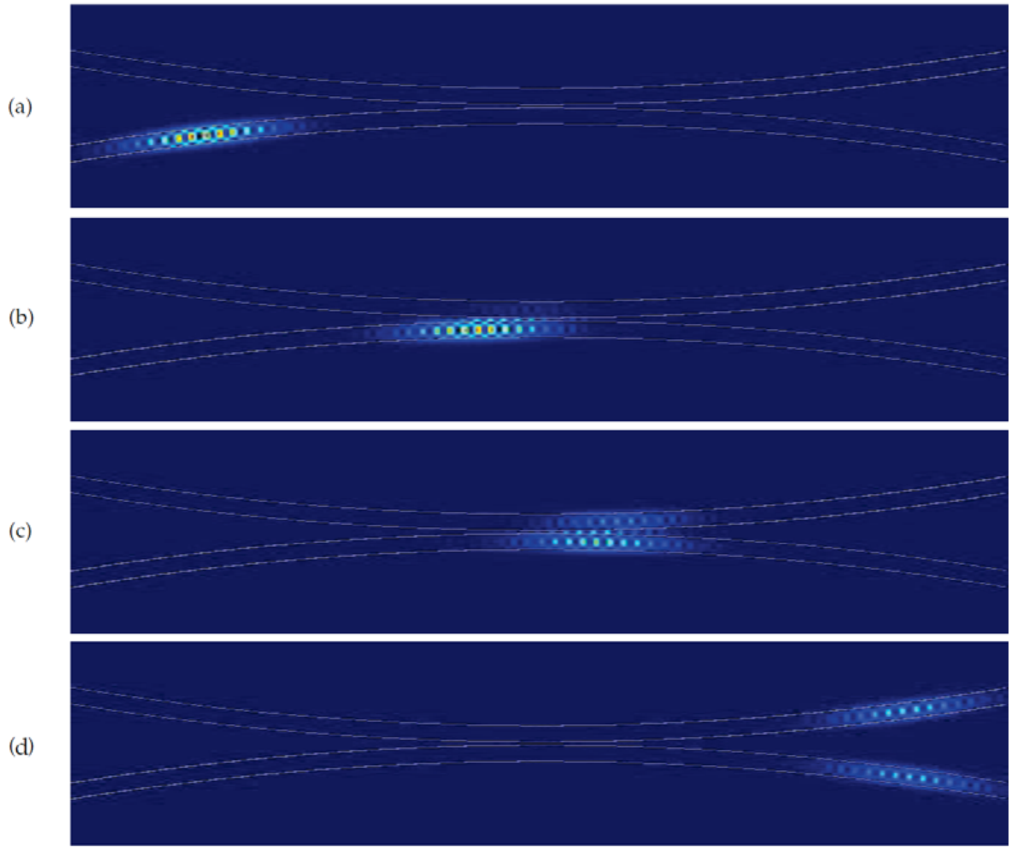

Supplementary Figure 5: Propagation of a pulse through the directional coupler. This example features waveguides with a bend radius of $71.6\mu m$ which is set to split the power of a signal 50%/50% to two output ports. The pictures are taken over a width of $23\mu m$ and a height of $5\mu m$. (a) The pulse starts at the lower left port. (b) When the pulse reaches the gap some of it leaks to the upper waveguide. (c) As the pulse continues, an additional part of the signal is leaked. (d) The split signal goes to the two output ports on the right.

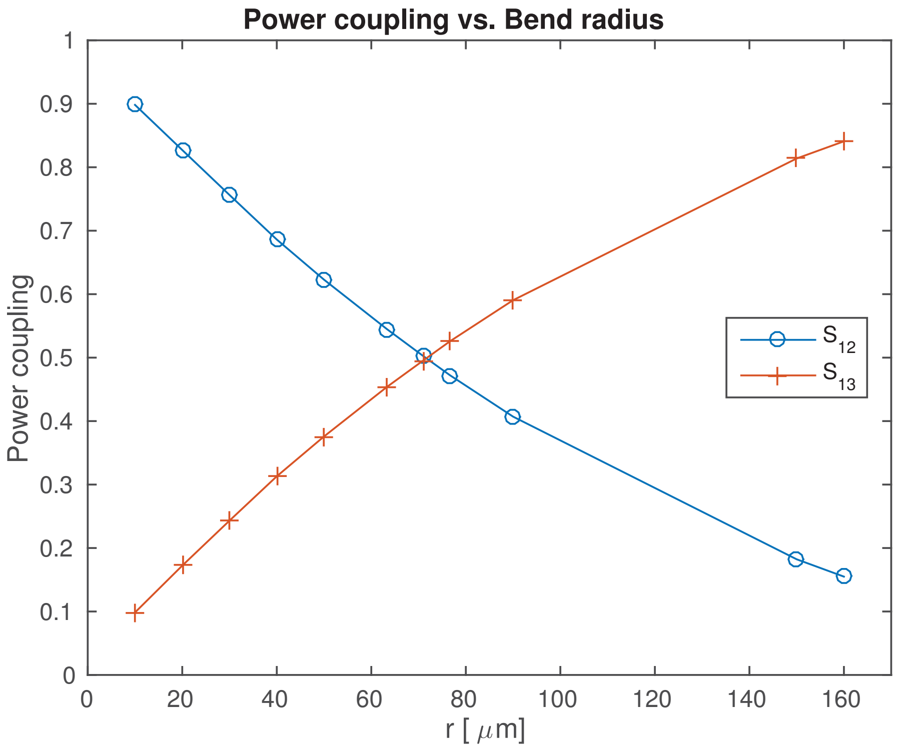

Supplementary Figure 6: Coupling values as a function of the bend radius. The value S_{12} represents the power that stays on the same bent waveguide while S_{13} represents the power coupled to the other bent waveguide. The gap was set to be $80nm$.

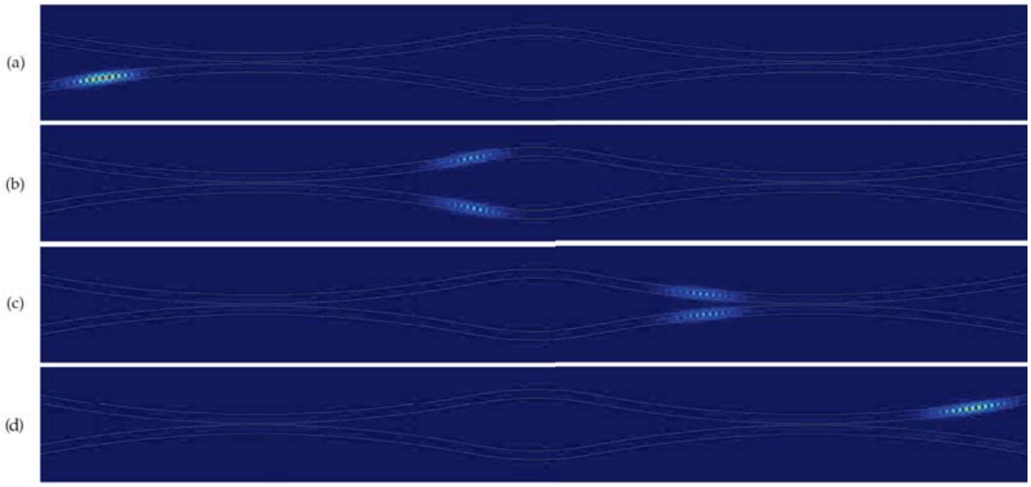

Supplementary Figure 7: Propagation of a pulse through the Mach-Zehnder interferometer built with two directional couplers. This example features two directional couplers introduced in Supplementary Figure 5. The two directional couplers are connected with bent waveguides with a bend radius of $20\mu m$. The pictures are taken over a width of $52\mu m$ and a height of $5.2\mu m$. (a) The pulse starts at the lower left port. (b) The pulse is split 50%/50% by the left directional coupler to two bent waveguides and introduced again to the second directional coupler. (c) The split signal approaches the second coupler. (d) The two split signals interfere over the second coupler to create one signal that propagates toward the upper right port, with no signal at the lower right port.

Supplementary note 1 - Light Propagating in a Waveguide

The electric field propagating in a waveguide satisfies the wave equation:

$$\nabla^2 \vec{E} + n^2 k_0^2 \vec{E} = 0$$

where $\vec{E}$ is the frequency-dependent electric field. When referring to modal propagation in a waveguide we may introduce a propagating constant β:

$$\vec{E}(x, y, z, \omega) = \vec{E}(x, y, \omega) \exp[i\omega t - i\beta(\omega) z]$$

for a Transverse Electric (TE) mode propagating along the z axis, and $\vec{E}(x, y, \omega)$ is the profile of the mode.

The propagation constant may be approximated with Taylor series over $\omega = \omega_0 + \omega'$ giving:

$$\beta(\omega) = \beta(\omega_0) + \left.\frac{\partial \beta}{\partial \omega}\right|_{\omega=\omega_0} \omega' + \frac{1}{2} \left.\frac{\partial^2 \beta}{\partial \omega^2}\right|_{\omega=\omega_0} \omega'^2 + \ldots = \beta_0 + \beta_1 \omega' + \frac{1}{2}\beta_2 \omega'^2 + \ldots$$

When propagating through a wave guide and considering only the first two terms, the first term β_0 will simply give a phase according to the wave guide's length, while β_1 will result in time delay of the signal:

$$E(t, z = L) = \exp[i\beta_0 L] E(t - \beta_1 L, z = 0)$$

The term β_2 is in charge of dispersion effects. For exaple, a Gaussian with a time width of T_0 is broadened into a Gaussian along the waveguide:

$$|E(t, z = L)|^2 = \frac{|E_0|^2}{\sqrt{1 + (\frac{\beta_2 L}{t})^2}} \exp\left[-\frac{t^2}{T_0^2[1 + (\frac{\beta_2 L}{t})^2]}\right]$$

where the last term neglected the phases gained by β_0 and β_1.

Slowly Varying Field

We can denote a complex envelope of the optical carrier as A:

$$E(z, t) = A(z, t) \exp[-iw_0 t]$$

where the envelope A changes much slower than the wave itself. Or, in other words, the typical times that define A are much longer than $\frac{2\pi}{\omega_0}$. We may obtain:

$$\frac{\partial A(t, z)}{\partial z} + i\frac{\beta_2}{2}\frac{\partial^2 A(t, z)}{\partial t^2} + \frac{\alpha}{2} A(t, z) = 0 \quad (1)$$

where α is denoting an absorption coefficient.

Non-Linear Effects

A non-linear effect may be needed as a building block for our design. Different non-linear optical effects can be observed with different materials. The different possibilities present different advantages and disadvantages.

In our case, we choose to work with the Kerr effect. The main advantage of the Kerr effect is that it is easily formulated mathematically. It is also commonly available on almost any material as opposed, for example, to the Pockels effect that is only available in non-centrosymmetric materials. However, the Kerr effect is a very weak effect and in order to observe a non-linear interaction, a very strong intensity is needed.

Stronger interactions may be available, such as the Pockels effect available in crystal materials such as lithium niobate, or specially designed poled polymers. Note, however, that the light-matter interaction is not as simple as the Kerr effect. Moreover, these materials demands additional design effort in order to exhibit desired parameters.

Non-Linear Kerr Effect

The Kerr effect describes the change of the refraction index in a material when an intensity of light is projected through it:

$$n = n_0 + n_2 I$$

Where I is the intensity. Equation 1 for A is rewritten introducing the Kerr effect:

$$\frac{\partial A(t,z)}{\partial z} + i\frac{\beta_2}{2}\frac{\partial^2 A(t,z)}{\partial t^2} + \frac{\alpha}{2}A(t,z) = i\gamma'|A(t,z)|^2 A(t,z)$$

Where γ' is related to n_2 by:

$$\gamma' \equiv \frac{n_2\omega_0}{cA_{eff}}$$

Where A_{eff} is defined by:

$$A_{eff} = \frac{\left[\int |E|^2 dxdy\right]^2}{\int |E|^4 dxdy}$$

Note that these equations were written such that $|A|^2$ is scaled to represent optical power (or W in MKS).

We can assume a single optical waveform with negligible dispersion ($\beta_2 = 0$), and get:

$$A(z,t) = A(z=0,t)\exp[-\frac{1}{2}\alpha z]\exp\left[i\gamma'|A(z=0,t)|^2\frac{1-e^{-\alpha z}}{\alpha}\right]$$

Next we may also neglect the absorption coefficient:

$$A(z) = A(z=0)\exp[i\gamma'|A|^2 z] \tag{2}$$

Equation 2 describes the electric field A as a physical value. As we are going to describe in the following sections, this electric field is going to represent logical and numerical values. In order to do so, we must scale the electric field to a unitless variable. We choose to scale the electric field according to an electric field amplitude E_1 that would represent the logical value "1". The scaled field will be represented by $A' = \frac{A}{|E_1|}$. The intensity of the field E_1 will be denoted by I_1.

Similarly, we would like to analyze the Kerr effect coefficient numerically, and we are going to scale it as well. Scaling of the non-linear coefficient involves the length, and the intensity scale factor of a given element. We may rewrite $\gamma' L I_1 \rightarrow \gamma$.

The scaling formulates the Kerr effect in a much simpler term:

$$A'(z=L) = A'(z=0)\exp[i\gamma|A'|^2]$$

We would like to use a very small γ in our design. However, we would like to find a typical value for γ that can be reasonable using available materials and typical sizes available for optical devices. We also can try to estimate a typical length L that will give a reasonably useful γ:

$$\gamma = \gamma' L I_1 A_{eff} = \frac{n_2\omega_0}{c} L I_1$$

A relatively high non-linear refractive index is observed over graphene-silicon samples measuring $n_2 = 4.8 \cdot 10^{-17} m^2/W$ [3]. A Gaussian mode in a circular single-mode waveguide will have an effective cross section of $A_{eff} = 2\pi R^2$ where R is the radius of the waveguide, typically $R = 4\mu m$, and a high power CW single mode source may exhibit a power of $P_0 = 300W$. A typical wavelength in the near infrared is $\lambda = 1\mu m$. Plugging in these values gives:

$$\frac{\gamma}{L} = 900 m^{-1}$$

Therefore, under these conditions a length of $L = 1.1mm$ gives $\gamma = 1$.

Also, the definition of this length may limit the time period that the system may work in. A typical time for a beam to travel through this medium will be:

$$t = \frac{nL}{c} = \frac{n}{n_2\omega_0 I_1}\gamma$$

Note that the non-linear refractive index derived [3] was calculated from an observation of four-wave mixing on a sample of graphene-silicon. The theoretical value calculated for this sample was a little higher, $n_2 = 7.7 \cdot 10^{-17} m^2/W$. It may be possible that further investigation of graphene-based samples may yield samples with even stronger interactions.

Supplementary note 2 - Energy-conserving transformations

Unitary transforms

A unitary transform is a transform that preserves the size of a complex vector. Usually, the transform is presented by a unitary matrix; that is, a matrix that when multiplied by a complex vector, the size of the result equals the size of the original vector. A unitary matrix U satisfies:

$$U^{\dagger}U = I$$

where the dagger sign represents the Hermitian Adjoint (conjugate and transpose) and I is the identity matrix.

If the vector elements represent electric fields then the size of the vector corresponds to the sum over all intensities, $\sum |E|^2$. In that case, a transform that preserves this size also conserves energy. The simplest example of an energy-conserving transformation is adding a phase by letting light propagate in a waveguide. The light that propagates gathers a phase to the electric field, however the size of the electric field does not change. This may be represented by a multiplication by $e^{i\phi}$ that does not change the size of the amplitude of the wave.

The definition of unitary transforms may be extended to a non-linear definition; that is, any transformation that maintains the size of the vector may be referred to as unitary transform. In our case, a phase induced by the Kerr effect still conserves the size of the electric field, however the phase depends non-linearly on the amplitude.

Directional Coupler

While adding a phase to the electric field is a unitary transform, we can't use that to manipulate more than one signal. Next we describe the optical directional coupler element that allows interaction of two signals. The basic

definition will start with a symmetric coupler which has a simple definition. This definition may be extended to a general linear unitary transform of two signals by adding the phase elements described above. This unitary transform will be represented by a 2×2 unitary matrix.

A symmetric directional coupling between two elements may be represented by a matrix:

$$\begin{pmatrix} E_1' \\ E_2' \end{pmatrix} = \begin{pmatrix} \rho & i\sigma \\ i\sigma & \rho \end{pmatrix} \begin{pmatrix} E_1 \\ E_2 \end{pmatrix}$$

In our case, these elements are electric fields propagating along two waveguides. Where E_1, E_2 represent incoming fields to the coupler and E_1', E_2' represent outgoing fields from the coupler. In order to have a conservation of energy the matrix must be unitary satisfying:

$$|\rho|^2 + |\sigma|^2 = 1$$

$$\rho\sigma^* = \rho^*\sigma$$

This coupling may be translated to any other energy-conserving transformation by choosing real $\rho = \cos\theta$, $\sigma = \sin\theta$ and adding a phase before and after the coupling. This gives:

$$\begin{pmatrix} E_1' \\ E_2' \end{pmatrix} = \begin{pmatrix} e^{i\phi_1} & 0 \\ 0 & e^{i\phi_2} \end{pmatrix} \begin{pmatrix} \rho & i\sigma \\ i\sigma & \rho \end{pmatrix} \begin{pmatrix} e^{i\psi_1} & 0 \\ 0 & e^{i\psi_2} \end{pmatrix} \begin{pmatrix} E_1 \\ E_2 \end{pmatrix} = T \begin{pmatrix} E_1 \\ E_2 \end{pmatrix}$$

We denote this transformation with T and we refer to T as a general representation of a unitary transformation matrix. A physical representation for this coupling is given in Supplementary Figure 1. Note that by choosing special cases of the phases it is possible to get a real unitary matrix that will be a rotation matrix, reflection matrix or a combination of the two.

Supplementary note 3 - Unitary Matrix Decomposition

After defining that a coupling between two channels may be represented by a 2×2 unitary matrix we can refer to a general array of couplers between N channels. We need to prove that any $N \times N$ unitary matrix may be decomposed to a series of transformations between two adjacent channels. These transformations will be denoted by T_k representing a transformation between channels k and $k+1$. We present a theorem and its proof while we also present the decomposition itself. This is done by inserting sets of $T_k^\dagger T_k = I$ as needed between multiplication terms.

Theorem 1. *A unitary transformation of n channels may be represented by $n \times n$ unitary matrix U. This matrix may be decomposed to $n(n-1)/2$ transformations between two adjacent channels.*

Assuming a column vector sized m where the first $k-1$ elements are zero:

$$V = \begin{pmatrix} 0 \\ \vdots \\ 0 \\ v_k \\ \vdots \\ v_n \end{pmatrix}$$

There may be a unitary matrix $T_k^\dagger(r_k)$, that multiplies V on the right and gives V' whose values are the same values of V except:

1. The k-th element turns to zero ($V_k' = 0$).
2. The $k+1$ element satisfies $|V_{k+1}'|^2 = |V_k|^2 + |V_{k+1}|^2$.

The parameter r_k, which defines the $T_k^\dagger$ matrix, is defined by the elements V_k and V_{k+1}, such that the conditions are satisfied. Notice that the matrix $T_k^\dagger$ is diagonal (with the value $t_{ii} = 1$) except the elements in rows and columns k and $k+1$ that hold a 2×2 matrix of the form described in the previous section.

The last column of a matrix U can be transformed into a column of zeros except for the last element as follows:

$$U = U \left[\prod_{i=1}^{n-1} T_i^\dagger(r_{i,n}) \right] \left[\prod_{i=n-1}^{1} T_i(r_{i,n}) \right] = U' \left[\prod_{i=n-1}^{1} T_i(r_{i,n}) \right]$$

Where U' is the transformed matrix after the last column is nullified except for the last element. Since U' is unitary, the size of this last element must be 1. We may assume that the last choice of T_{n-1} also ensures that this element equals 1 by adding an appropriate phase. This also means that the other elements in the last row of U' are zero.

We can continue with this process for the $n-1$ column of U' and nullify its elements except the diagonal element. We may continue with all other

columns until the transformation gives us the identity matrix. The other side of the equation is left with the decomposition.

This decomposes U as follows:

$$U = \prod_{j=1}^{n-1} \left[\prod_{i=j}^{1} T_i(r_{i,j+1}) \right]$$

An example of five-channel coupling is given in Supplementary Figure 2. This setup presents ten couplings between adjacent channels. Note that each coupling is a coupling of the sort presented in Figure 1.

Supplementary note 4 - Convergence

As described in the main article we try to fin solutions to:

$$\begin{pmatrix} B \\ F \end{pmatrix} = M \begin{pmatrix} A \\ F' \end{pmatrix} \tag{3}$$

Solutions to equation (3) are equilibrium states that will hold the output as long as the input is preserved. However, these solutions are not necessarily stable. Also, when changing the input there is no guarantee that the system will converge to the desired state. Moreover, the system may be unused and wake up in any arbitrary state. There is no guarantee that the system will converge given a new input.

However, there is a way to ensure convergence. This is done by defining the dependencies of the F feedback channels. Unfortunately, we may not define F as a direct function of the input channels. But it is possible to define every element of F as dependent on the inputs and all the values in the rows above it; this is done by choosing $M_{jk} = 0$ for $j > m$ and $k \geq j$ where m is the number of rows in A or B. This will give the following:

$$F_{ij} = \sum_{k=1}^{m} M_{(i+m)k} A_{kj} + \sum_{k=1}^{i-1} M_{(i+m)(k+m)} F'_{kj}$$

While F'_{kj} is dependent only on F_{kj}.

Notice that the diagonal elements of $M_{ii} = 0$, where $i > m$. This corresponds to the elimination of the feedback of a channel to itself. In other words, this eliminates the self-resonance for the values of F; this means that

there is no amplification of F due to resonance. It may be possible to design a system with self-amplification for the feedback channel, and this way lower the needed γ while shortening the length L of the feedback loop. However, the convergence to the resonance value will take a longer amount of time, such that the overall time will be the same, if not longer.

Convergence Time Period

Note that under the assumption that all the feedback channels converge one after the other, the overall convergence time is the sum of the times for all the channels:

$$T = \sum_i t_i = \frac{n}{c} \sum_i L_i = \frac{n}{n_2 \omega_0 I_1} \sum_i \gamma_i$$

We may plug in the values presented in section and choose $n = 2$, which gives:

$$T = 7.4 \cdot 10^{-12}[s] \cdot \sum_i \gamma_i$$

Therefore, we would like to minimize the sum to get a low convergence time and, as a result, high working frequency of the device.

Since we use continuous waves, energy continues to flow through the system while converging. In a sense, this energy is wasted until a steady state is achieved. However, this wasted energy is not translated to heat the system, in fact, it is actually cleared from the system in an optical manner through the outputs. This converging energy may be assessed:

$$E_c = I_1 A_{eff} T = \frac{n A_{eff}}{n_2 \omega_0} \sum_i \gamma_i \approx 2.2 \cdot 10^{-9}[J] \sum_i \gamma_i$$

Note that this size became independent of the intensity I_1. Lower intensity would mean longer length of the non-linear element L, longer propagation time t and convergence time T such that E_c keeps its value. Note that this size may be used as a quality factor of the device, defined only by typical dimensions and values of waveguides.

Supplementary note 5 - Further Reduction

There may be some ways to simplify the decomposition of M to adjacent coupling matrices.

Lowering the Rank

Lowering the rank of M or its dimensions directly leads to a simpler design of the decomposition.

We may find relations that lower the number of F channels needed. For example, notice the bottom rows on the A and B matrices. There may be a possibility to include these rows as part of the F' and F matrices and look for their inverse.

In general, the rank of matrices $\begin{pmatrix} A \\ F' \end{pmatrix}$ and $\begin{pmatrix} B \\ F \end{pmatrix}$ may even be lower than the number of states (or the number of columns). In other words, it may be possible to choose carefully the values of F', F and M that satisfy the conditions, while the size of M is smaller than the number of states.

Sparse Matrix

We may find an M matrix that holds the value 0 on some of its elements. We may consider different cases.

The matrix M may have zeros on its upper right triangular. We may remember that the decomposition turns the elements of the vectors to zero from the top or the bottom towards the diagonal. If an upper right diagonal has zeros, it reduces the number of adjacent decomposition since these elements are already zero. In fact, it is possible to prove that this type of matrix may eliminate the use of feedback loops. The number of non-zero elements on the first row determines the number of channels that are used. The number of couplings between these channels is limited by the dimension of the matrix.

The matrix M may be a block matrix. Each block may represent a node with fewer channels to couple. For example, we consider a 5×5 matrix that may be decomposed to 3×3 and 2×2 matrices. In this example the number of adjacent matrix decompositions is reduced from 10 to 4.

Next, we may refer to two cases. First we consider the blocks are set on the main diagonal:

$$M_5 = \begin{pmatrix} M_3 & 0 \\ 0 & M_2 \end{pmatrix}$$

If one of those matrices refers only to F channels, that means that those channels are self-defined and are independent from the rest of the system. In that case, those channels may be removed from the system.

X_1	X_2	Y_1	Y_2
0	0	0	0
0	1	1	1
1	0	1	0
1	1	0	1

Table 1: A truth table of the reversible XOR gate. X_1 and X_2 represent two input bits, while Y_1 and Y_2 represent two output bits.

We also consider that the blocks are set on the secondary diagonal. For example:

$$M_5 = \begin{pmatrix} 0 & M_3 \\ M_2 & 0 \end{pmatrix}$$

Here we may consider two nodes that are interconnected in a cascade fashion.

Supplementary note 6 - Representations for XOR Gate

The XOR gate is defined by the truth table given in Table 1.

The output Y_1 produces the XOR while the output Y_2 gives the same value of X_2, unchanged. We may exchange the inputs and outputs of the gate and get the same behavior of the gate.

Note that the number of zeros and ones is not preserved for each state. For example, the second state (second row of Table 1) shows that the input includes an input of one bit with the value 1 and another with the value 0 and the output includes two bits with the value 1.

This may limit our representation of states. If the possible values of a bit 0 and 1 are represented by an electric field E_0 and E_1 respectively, and $|E_0|^2 \neq |E_1|^2$, the energy may not be conserved in that state. That may be solved in two possible representations, "Phased bit", and "Doubled bit".

Phased Bit

We may still use one channel where the difference between the states is introduced in the phase such that $E_1 = 1$ and $E_0 = e^{i\phi}$. Note that the field is scaled such that $|E_0|^2 = |E_1|^2 = 1$. We may choose the phase difference such that $E_0 = -1$.

This representation may raise a problem since an additional phase is added as the light propagates in time. That means that the device may not differentiate between $(0,0)$ state and the $(1,1)$, and between $(1,0)$ and $(0,1)$ states. A reference is needed in order to allow the desired differentiation. For example, another channel with input and output that is always 1 may serve as a reference in the system.

Doubled Bit

Another approach to overcome loss of energy because of the representation issue is to represent each bit with two channels that hold two possible values E_0 and E_1, and if one channel holds one of the values the other channel holds the other value. In other words, one channel holds a value and another channel holds the negation of this value. We may choose $E_0 = 0$ and $E_1 = 1$.

This will mean that the 0 bit is represented with:

$$|0\rangle = \begin{pmatrix} 0 \\ 1 \end{pmatrix}$$

and the 1 bit is represented by:

$$|1\rangle = \begin{pmatrix} 1 \\ 0 \end{pmatrix}$$

The energy needed to represent the two states is the same and it is $|E_0|^2 + |E_1|^2 = 1$.

Phased Bit Solution for the XOR Gate

In the phased bit representation of states the input matrix is:

$$A = \begin{pmatrix} -1 & -1 & 1 & 1 \\ -1 & 1 & -1 & 1 \\ 1 & 1 & 1 & 1 \end{pmatrix}$$

The output matrix is:

$$B = \begin{pmatrix} -1 & 1 & 1 & -1 \\ -1 & 1 & -1 & 1 \\ 1 & 1 & 1 & 1 \end{pmatrix}$$

C	X_1	X_2	C'	Y_1	Y_2
0	0	0	0	0	0
0	0	1	0	0	1
0	1	0	0	1	0
0	1	1	0	1	1
1	0	0	1	0	0
1	0	1	1	1	0
1	1	0	1	0	1
1	1	1	1	1	1

Table 2: A truth table of the reversible Fredkin gate. X_1, X_2 and C represent three input bits, while Y_1, Y_2 and C' represent three output bits.

Where the first row of A is transformed by the XOR operation to the first row of B. The second row of A remains the same as the second row of B. The third row that represents the reference is also the same on A and B.

Next, we need to find the F matrix that satisfies the conditions.

$$B^\dagger B - A^\dagger A = \begin{pmatrix} 0 & -2 & 0 & 2 \\ -2 & 0 & 2 & 0 \\ 0 & 2 & 0 & -2 \\ 2 & 0 & -2 & 0 \end{pmatrix} = F'^\dagger F' - F^\dagger F$$

As explained earlier, the diagonal of $B^\dagger B - A^\dagger A$ is zero because of the conservation of energy. Note that since this result does not equal 0 in all the matrix elements, this serves as a proof that under this representation non-linear manipulation is obligatory. The next step is to find a unitary matrix M and γ values that satisfy equation (3).

Supplementary note 7 - Representations for Fredkin Gate

The Fredkin gate is defined by the truth table given in Table 2.

The output satisfies $Y_1 = X_1$ and $Y_2 = X_2$ if $C = 0$. Also, $Y_1 = X_2$ and $Y_2 = X_1$ if $C = 1$. Note also that $C' = C$.

The Fredkin gate is special because of the fact that the number of zeros and ones is preserved for each state as opposed to other reversible gates, such

as the reversible XOR. This permits freedom in choosing our representation of states. The possible values for the bits 0 and 1 may be represented by any electric field E_0 and E_1, as long as they are different.

Binary Bit

We are going to use straightforward representations for the bit values where $E_1 = 1$ and $E_0 = 0$.

Note that in this representation, the state $(0, 0, 0)$ may be omitted since it is trivial, where no energy enters the device and no energy exits the device. This will lower the number of states we have to deal with to 7 instead of 8, while the $(0, 0, 0)$ state is ignored in our discussion.

Binary Bit Solution for the Fredkin Gate

In the binary bit representation of states the input matrix is:

$$A = \begin{pmatrix} 1 & 0 & 1 & 0 & 1 & 0 & 1 \\ 0 & 1 & 1 & 0 & 0 & 1 & 1 \\ 0 & 0 & 0 & 1 & 1 & 1 & 1 \end{pmatrix}$$

The output matrix is:

$$B = \begin{pmatrix} 1 & 0 & 1 & 0 & 0 & 1 & 1 \\ 0 & 1 & 1 & 0 & 1 & 0 & 1 \\ 0 & 0 & 0 & 1 & 1 & 1 & 1 \end{pmatrix}$$

Next we need to find the F matrix that satisfies the conditions.

$$B^\dagger B - A^\dagger A = \begin{pmatrix} 0 & 0 & 0 & 0 & -1 & 1 & 0 \\ 0 & 0 & 0 & 0 & 1 & -1 & 0 \\ 0 & 0 & 0 & 0 & 0 & 0 & 0 \\ 0 & 0 & 0 & 0 & 0 & 0 & 0 \\ -1 & 1 & 0 & 0 & 0 & 0 & 0 \\ 1 & -1 & 0 & 0 & 0 & 0 & 0 \\ 0 & 0 & 0 & 0 & 0 & 0 & 0 \end{pmatrix} = F'^\dagger F' - F^\dagger F$$

As explained earlier, the diagonal of $B^\dagger B - A^\dagger A$ is zero because of the conservation of energy. Note that since this result does not equal 0 in all the matrix elements, this serves as a proof that under this representation, non-linear manipulation is obligatory. Again, the next step is to find a unitary matrix M and γ values that satisfy equation (3).

Supplementary note 8 - Fredkin Gate Numerical Solution

The numerical solution for the Fredkin gate was done in several steps.

A solution for equation (3) was found where M is an 8×8 matrix. The matrix found was actually a block matrix where $M_{ij} = 0$ for $i \leq 3, j \leq 5$ and for $i > 3, j > 5$. In a matrix form M is defined by blocks:

$$M = \begin{pmatrix} 0 & M_3 \\ M_5 & 0 \end{pmatrix}$$

where M_3 is a 3×3 matrix and M_5 is a 5×5 matrix. This means that the design of the device may be reduced to 2 nodes, which simplifies the coupling array. This design is presented in Supplementary Figure 3.

Note also that M_5 has zeros on the three elements of the upper right triangular parts. This, in turn, also saves three adjacent couplings on its decomposition. This M_5 matrix may be decomposed with seven adjacent couplings, and the M_3 matrix may be decomposed using three adjacent couplings. Overall, a design with 10 couplings may be used. In fact, this special solution for M_5 also eliminates the need for feedback loops and less non-linear elements are needed while the same operation may be performed by several smaller nodes as described in Figure 2. Nodes M^1, M^2 and M^3 in Figure 2 replace node M_5 in Supplementary Figure 3, while node M^4 in Figure 2 is equivalent to node M_3 in Supplementary Figure 3.

In the configuration suggested, the feedback channels are eliminated and instead, a unidirectional flow of optical signals is observed between the nodes. The first non-linear channel is dependent only on the inputs, so the values of F_1 and F_1' converge after light propagates through it. Similarly, the next non linear elements are dependent only on the signals arriving to the previous node, and their values F_i and F_i' converge as soon as all appropriate signals have reached the previous node.

The elimination of the feedback allows the usage of pulses instead of CW. While a feedback channel may requires a relaxation period, it may only be implemented with CW. When feedback is eliminated we may use pulses by making sure that all signals reach the appropriate node at the same time, and a careful design of the optical path is needed.

The simplification of the device also modifies the term for the convergence time. The sum of all γ coefficients is now replaced by:

$$S_\gamma = \gamma_1 + \gamma_2 + \gamma_3$$

And the convergence time is:

$$T = t_1 + t_2 + t_3 = \frac{n}{n_2 \omega_0 I_1} S_\gamma$$

Numerical results trying to minimize S_γ produce a matrix with $S_\gamma = 12.5$. This value will be used later for an assessment of the convergence time.

The numerical solution that was found serves as a solution for the theoretical problem giving an M matrix and a γ vector. Note that the fact that a solution was found at all is the first step in proving the feasibility of the production of an optical Fredkin gate. It proves that, at least theoretically, a Fredkin gate may be produced only with linear coupler elements, linear phase elements, and non-linear energy-conserving elements.

Supplementary note 9 - Optical elements simulations

Directional Coupler

The simulations of the directional coupler were performed based on the template presented in Ref. [2]. Two circular waveguides were used to create a four-port network. The inner and outer radii differ by the width of the waveguide chosen. The two waveguides come very close to each other and create a very small gap which allows coupling between the two waveguides. In our tests, we chose a gap of $80nm$ when calculating the transmission coefficients from one port to the other ports while changing the radii of the circular waveguides. A schematic design is given in Supplementary Figure 4.

Note that since there is symmetry between all four ports, we can run a simulation with a source at one port and there is no need to rerun the same simulation with the other ports. An example for a simulation of the directional coupler is given in Supplementary Figure 5. The simulation shows a short pulse starting at the lower left port and propagating along the lower bent waveguide. When the pulse reaches the gap, some of it leaks to the upper waveguide and finally the signal is split between the two output ports on the right. Note that reflected signals are invisible and practically negligible.

The results show a loss lower than 0.3% in the energy, or $-0.013db$. A graph showing different coupling results as a function of the bend radius is given in Supplementary Figure 6. When the waveguide bend radius chosen is $71.6\mu m$ the coupling showed a 50% energy transmission to the two output ports.

Mach-Zehnder interferometer

The directional coupler may be used as a basic element for a Mach-Zehnder interferometer (MZI). This is done by using two of these elements and introducing a phase difference in one of the channels connecting the two directional couplers. An example for a simulation of a Mach-Zehnder interferometer is given in Supplementary Figure 7. In this example, two directional couplers are connected with two bent waveguides with a bend radius of $20\mu m$. This case features no phase difference between the channels. Phase difference may be introduced by enlarging the bend radius of one of the added bent waveguides to shorten its optical length. This device still exhibits a very low power loss that is calculated to be lower than 0.3%.

Kerr Element

The simulation of the Kerr Element used the same waveguide, where a thin graphene layer is infused centered over the silicon waveguide. It was centered at a height of $z = 110nm$. While the actual thickness of a graphene sheet would be $0.3nm$, the thickness chosen for the simulation was $\Delta z = 1nm$, for two reasons. First, it is possible to consider the interaction with three layers of graphene. As measured in Ref. [5], the number of layers simply means a linearly stronger phase shift. Second, an FDTD simulation has to mesh simulation points along the thickness of the sample, which will require time and computing resources. Also, a thinner layer will require stronger non-linear interactions which may interfere with the FDTD simulation and the solution for this simulation might diverge and give unexpected results.

An illustration of the cross section was given in Figure 3. The length chosen for the simulation was $L = 5\mu m$. The monitors were placed $0.5\mu m$ before and after the Kerr element, trying to check if there is any loss or reflection due to mode mismatch. The non-linearity coefficients were chosen as $\chi^{(1)} = 9$ and $\chi^{(3)} = 2.095 \cdot 10^{-15}[m^2V^{-2}]$ [4, 5]. Several amplitudes were tested trying to determine loss and phase response to the power.

The results show a negligible loss, lower than 0.07% in the energy, or $-0.003db$. The phase response is given in Figure 4. It shows a relatively high response giving:

$$\frac{\gamma}{L} = 6.31 \cdot 10^3[W^{-1}m^{-1}]$$

As explained earlier, a sum of the non-linear values was calculated as

$S_\gamma = 12.5$. This result corresponds to a length of $L = 2mm$ and a converging time of $T = 2 \cdot 10^{-11}s$ when using $1W$ sources. A convergence energy $E_c = 2 \cdot 10^{-11}J$. The convergence time is suitable for systems with frequency higher than $100GHz$. Note of course that a higher system frequency may be achieved with higher power and designing shorter elements. Also, low power devices may be produced with longer elements at the expense of the system frequency.

Although we regard the convergence energy as energy loss, remember that this loss is not dissipated as heat but rather is removed from the system through the output channels. A careful design may redirect the output to be reused while converging. This recycling process may be used, for example, as additional pumping needed for excitation and population inversion of a laser source. This source may be the source for the system, thus the recycling process reduces its power consumption.

Supplementary note 10 - Settings and parameters

When we deal with the available building blocks, we have to remember that we want to build devices with negligible loss. In other words, the sum of the intensity over all inputs should be nearly the same as the sum of the intensity over all outputs.

Note that negligible loss is possible to maintain providing the advances in fabrication. An "Ultra-high-Q" optical resonator has been presented [1], featuring a design and fabrication process that managed to produce a toroid microcavity resonator on a chip with a Q factor in excess of 100 million. This suggests that loss is negligible and was eliminated by the advanced fabrication methods described.

The formalism presented deals with an ideal theory behind the device. The assumptions presented below only refer to our theory. In the results section, we tested different optical elements, to challenge these assumptions. We tested the feasibility of physically implementing this theory and ultimately demonstrated that these assumptions are almost entirely correct. The assumptions regarding the behavior of the device are as follows:

- *Negligible absorption.* The device and each of its elements have no absorption, or the absorption is negligible.

- *Single monochromatic continuous wave.* We assume a continuous wave (CW) in a single wavelength that propagates through the device. This

may happen after a long time when the device reaches a stable state. This will eliminate the time dependency of the intensity envelope, such that the dispersion is also eliminated. Note that it is possible to modify the device to handle pulse signals by making sure that when interaction is needed between several signals, they arrive at the same point in space and time. A pulse which is longer than the wavelength by an order of magnitude, may be long enough to neglect the effects of dispersion-like interferences, yet short enough to exhibit very high bandwidth compared to conventional electronic circuits.

- *Unidirectional light propagation.* Light propagates in one direction over each channel. We assume that all couplings exhibit zero reflection. Though bidirectional propagation may reduce the design complexity of the device, it may complicate the analysis, especially if non-linearities are involved.

- *Lossless Kerr effect.* The Kerr effect may only manipulate the phase and not the amplitude. This is possible only if the non-linear coefficient is a real number (not a complex or imaginary number).

- *As linear as possible.* We would like to avoid using non-linear effects on the design as much as possible and prefer to use linear elements that can take their place. Only when no linear element may be found to fulfill a specific need, Non-linear elements will be used to patch and manipulate the light signals. Linear analysis will be done to determine those needs.

- *Scaling.* Since only one wavelength is involved, scaling is possible where geometric lengths may be part of the physical properties of the elements. This way a light with a wavenumber k propagating in a waveguide with a length L gathers a phase ϕ. In order to simplify our expressions we will use $kL \to \phi$. This phase may be controlled by manipulating both k and L.

  We can also scale the electric field. We are going to give certain electric fields logical values. The scaled electric field has no units. It may hold a complex number, but typically it holds integer values, for example, $E_1 = 1$, $E_0 = 0$. Note, however, that these fields represent a real field with real intensity or power, given by the scale factor.

Similarly, we may define scaling of the Kerr effect. A phase induced by the Kerr effect will be $\phi = \gamma|E|^2$, where γ is the scaled Kerr coefficient and E is the scaled electric field. Note that both E and γ values have no units. Note that γ is linearly dependent on the length of the Kerr medium and may be manipulated by choosing the right materials and proper lengths for the design.

Supplementary References

[1] D. Armani, T. Kippenberg, S. Spillane, and K. Vahala. Ultra-high-q toroid microcavity on a chip. *Nature*, 421(6926):925–928, 2003.

[2] L. Chrostowski and M. Hochberg. *Silicon photonics design*. Lulu. com, 2013.

[3] T. Gu, N. Petrone, J. F. McMillan, A. van der Zande, M. Yu, G.-Q. Lo, D.-L. Kwong, J. Hone, and C. W. Wong. Regenerative oscillation and four-wave mixing in graphene optoelectronics. *Nature Photonics*, 6(8):554–559, 2012.

[4] M. L. Nesterov, J. Bravo-Abad, A. Y. Nikitin, F. J. García-Vidal, and L. Martin-Moreno. Graphene supports the propagation of subwavelength optical solitons. *Laser & Photonics Reviews*, 7(2):L7–L11, 2013.

[5] H. Zhang, S. Virally, Q. Bao, L. Kian Ping, S. Massar, N. Godbout, and P. Kockaert. Z-scan measurement of the nonlinear refractive index of graphene. *Optics letters*, 37(11):1856–1858, 2012.
